# Oxidative stress, NOx/l-arginine ratio and glutathione/glutathione S-transferase ratio as predictors of ‘sterile inflammation’ in patients with alcoholic cirrhosis and hepatorenal syndrome type II

**DOI:** 10.1080/0886022X.2018.1459699

**Published:** 2018-04-16

**Authors:** Vanja P. Nickovic, Dijana Miric, Bojana Kisic, Hristina Kocic, Marko Stojanovic, Salvatore Buttice, Gordana Kocic

**Affiliations:** a Clinical Hospital Center of Pristina, Laplje Selo, Serbia;; b Faculty of Medicine, University of Pristina, Kosovska Mitrovica, Serbia;; c Medical Faculty, University Maribor, Maribor, Slovenia;; d Faculty of Medicine, University of Nis, Nis, Serbia;; e Department of Urology, San Giovani di Dio Hospital, Agrigento, Italy

**Keywords:** Oxidative stress, nitrites (NOx), liver cirrhosis, renal failure, hepatorenal syndrome

## Abstract

Continuous intake of alcohol leads to liver cirrhosis because of imbalance of oxidative stress/antioxidative defense and chronic ‘sterile inflammation’. Hepatorenal syndrome (HRS) is the most severe complication of liver cirrhosis. The aim of our study was to assess: (1) the oxidative stress/antioxidative defense markers such as malondialdehyde (MDA), oxidative glutathione (GSH) and glutathione S-transferase (GST), (2) inflammation [C-reactive protein (CRP)], and (3) nitrate/nitrite levels (NOx) and its substrate L-arginine level. The study enrolled three groups: a group with cirrhosis and HRS (48 patients), a group with cirrhosis without HRS (32 patients), and a control group (40 healthy blood donors). All the patients with cirrhosis and HRS had type II HRS. MDA concentration was significantly higher in the groups with cirrhosis with and without HRS. Significant positive correlation was documented between the MDA level and de Ritis coefficient (AST/ALT), a marker of liver damage severity; between MDA and inflammation (CRP); between MDA and NOx concentration in the groups with cirrhosis with and without HRS. The correlation between MDA and creatinine level was significant in the group with HRS. The levels of GSH and GST were significantly lower in the groups with cirrhosis with and without HRS. The results of the study revealed that an increase in MDA and NOx concentration, along with decreased values of antioxidative defense and L-arginine, may indicate that liver damage can have an influence on progression to renal failure.

## Introduction

Hepatorenal syndrome (HRS) is a potentially reversible syndrome that develops in chronic liver failure. It is a functional and progressive impairment in renal function. The causes of its onset are numerous, such as advanced hepatic failure and portal hypertension, worsening splanchnic vasodilation, as well as circulatory vasoconstrictors increase. HRS is characterized by changes in systemic circulation due to increased activity of the endogenous vasoactive mechanisms. Renal failure is a consequence of a severe and progressive constriction of blood vessels and a decreased glomerular filtration rate (GFR) [1]. In end-stage liver cirrhosis, precipitating factors such as infections, shock, hemorrhage, and paracentesis may also contribute to the development of HRS [2]. Based on a recent consensus on definition, diagnosis and therapy of HRS, the International Ascites Club has established a new criterion on HRS diagnosis [3]. Renal failure may develop rapidly with a serum creatinine level >220 μmol/l and is associated with type I HRS, or it may develop slowly with a serum creatinine level greater than 130 μmol/l, being associated with type II HRS. These two types of HRS are two different clinical patterns of renal failure manifestation [4,5].

Chronic alcohol consumption causes oxidative liver damage. Ethanol intoxication is the cause of an increased intestinal permeability and endotoxemia. Endotoxins can activate Ito cells to secrete collagen and other extracellular matrix components through TGF-β growth factor of Kupffer cells [6]. Oxidative damage is the result of abnormal production of reactive oxygen species (ROS), toxic effects of ethanol and its metabolites and the effects of activated phagocytes. Also, continuous use of ethanol intensifies ROS and lipid peroxides production. ROS may be produced in a number of enzyme systems such as mitochondrial electron transport chain, alcohol-inducible cytochrome P450 (CYP 4502E1), microsomal mono-oxygenase system, microsomal NADPH oxidase, cytosol xanthine, and aldehyde oxidase [7]. Lipid peroxidation (LP) represents the most important progrediant and in the most cases irreversible mechanism of parenchymal liver ROS damage. LP of polyunsaturated fatty acids in membrane lipids and subcellular organelles results in delipidation of membrane lipoprotein lipid bilayer, along with alteration of its structure and function, as well as enzymes inactivation [8]. Damaged hepatocytes release molecules associated with tissue damage (DAMP). As a result, the process of sterile inflammation develops [9]. Released DAMP molecules as molecules with pro-inflammatory characteristics induce C-reactive protein (CRP) synthesis, fibrinogen and other proteins of the acute inflammation phase in the remaining hepatocytes [10].

Series of complex LP reactions develop products that consist of carbonyl groups, such as 4-hydroxyzine and short-chained malondialdehyde (MDA). MDA is a highly reactive secondary product of LP that reacts with SH and amino groups of phospholipids, proteins, peptides and nucleoids. Reaction of MDA with thiol groups of membrane enzymes produces inactivation of membrane enzymes, while MDA oxidation with nucleic acids develops mutation and liver cancerogenesis [11]. Peroxynitrates and lipid peroxide by reactions of nitroxidation and nitrosation modify biomolecules by inducing change in their structure and function [12]. This is followed by an irreversible damage of hepatocytes and dysfunction of vascular endothelium [13]. With progression of a chronic liver and renal failure, an increased production of reactive species and MDA exceeds liver capacity for synthesis and regeneration of the necessary amounts of reduced glutathione and glutathione of dependent enzymes. Glutathione may directly react with and degrade oxidative stress products: hydroperoxides, lipid peroxides, and aldehydes [14]. Glutathione as a coenzyme for glutathione dependent enzymes, glutathione S-transferase (GST), and glutathione peroxidase (GPX), as well as glutathione reductase, is indirectly responsible for detoxification of toxic endogenous and exogenous compounds. Glutathione maintains cellular integrity as well as physiological processes and signals using AO system. GST and GPX protect nuclear membrane from lipid peroxides. GST can be found in the liver, kidneys, erythrocytes, leukocytes, testicles, and placenta, with the highest level of GST liver isoenzyme. GSTs are involved in the detoxification of electrophilic cytotoxic compounds (such as oxidatively modified proteins, lipids, carbon hydrates and nucleic acid) and carcinogenic compounds as well. Electrophilic benzopyrene that may induce carcinogenesis is activated by cytochrome P450. Oxidized benzopyrene results in guanine damage in hepatocyte DNA. Glutathione S-transferases are able to detoxify benzopyrene metabolite by conjugation reactions [15].

Zuwała-Jagiełło et al. [16] reported that lipid peroxides and peroxynitrites play a key role in the pathogenesis of alcohol-induced liver disease. They postulate that in conditions of chronic oxidative stress and inflammation, MDA and nitrates together with other oxidative protein and carbohydrate molecules (AOPP and AGE) participate in the pathogenesis of abnormalities in chemodynamics of liver and kidneys and in the progression of renal failure [17].

Our previous studies showed that arginine metabolites levels, such as ADMA and SDMA, may correlate with the degree of renal failure and the development of HRS in patients with alcohol-induced decompensated liver cirrhosis [18]. Accumulation of ADMA inhibits NO synthesis, causing vasoconstriction of the renal blood vessels, reduction of glomerular filtration, and retains SDMA in the kidney. Structural and functional hepatic impairments associated with fibrosis progression result in a progressively increased portal vessels resistance that triggers splanchnic vasodilation development [19]. In cirrhotic liver, a deficient NO vasodilatory effect and increased vasoconstrictive effects of endothelin and angiotensin may aggravate portal hypertension. A vasodilation of arterial splanchnic vessels with reduced NO production in the liver, as well as prostacyclin, glucagon, and adrenomedullin effects, induces a decrease in mean arterial pressure [20]. In patients with decompensated cirrhosis, abnormalities in the circulation are responsible for the progression of portal hypertension and the development of refractory ascites and HRS [21]. Cumulative oxidative stress and inflammation result in endothelial function damage and hemodynamic imbalance, as well as irreversible liver and kidney failure and the development of vascular collapse, mediated by cytokines of hepatic macrophages and neutrophils, iNOS and peroxynitrates activities, and decreased activity of AOS [22].

The mechanisms with which oxidative stress and inflammation contribute to the toxic effect of alcohol and its metabolites and the development of chronic liver and renal damage have not been fully explained up to now. Considering the fact that free radicals have very short half-lives, clinical evaluation of oxidative stress is based on the measurement of stable oxidized compounds (MDA). Hypothesis is based on the expected difference in MDA concentration in cirrhosis patients with or without HRS. Accordingly, the purpose of this investigation was to evaluate the level of MDA in plasma of patients with cirrhosis with or without HRS as well as to investigate the importance of MDA in patients with alcohol-induced cirrhosis in relation to the severity of liver disease and the level of HRS development.

## Patients and methods

The study was a retrospective one conducted from March 2016 to February 2017. It comprised two groups of subjects: a group with cirrhosis and a group of healthy subjects. Patients with cirrhosis were hospitalized in Gastroenterology Ward in the Nis Clinical Center. The cirrhosis group comprised 80 patients who had been consuming alcohol for over 10 years. All of them were male subjects, aged between 25 and 70 years. Average age was 53.21 ± 11.75 years. All patients were in the end-stage alcohol-induced cirrhosis with a moderate to severe ascites. The group was divided according to the HRS presence. HRS was diagnosed in 48 patients with cirrhosis and it was not diagnosed in 32 patients. All the patients with cirrhosis and HRS had type II HRS. The patients with HRS had indwelling catheters. The control group comprised 42 patients who were volunteer blood donors from the Blood Transfusion Ward in the Niš Clinical Center. All of the control group subjects were male subjects, average age was 51.13 ± 23 years with normal laboratory values.

During this investigation, it was also very important to determine diagnosis of cirrhosis based on a clinical biochemical and ultrasound findings. In addition, diagnosis of cirrhosis was determined with biopsy of the liver. The presence of ascites was confirmed with diagnostic paracentesis. HRS was diagnosed in accordance with the latest criteria as suggested by the International Ascites Club [3]. Criteria comprised cirrhosis with ascites, low glomerular filtration, creatinine value in serum greater than 133 μmol/l (larger than 1.5 mg/dl), proteinuria less than 500 mg/daily, absence of shock, absence of bacterial infection, loss of liquid, poor renal function after discontinuation of diuretic therapy (the value of creatinine in the serum that was sustained at least 48 h on the level ≥133 μmol/l after application of albumin in a dose from 1 to 100 g/kg per day), treatment without nephrotoxic drugs and absence of parenthetic renal disease (a patient did not have proteinuria >500 mg/daily, no microhematuria >50 erythrocytes, as well as the absence of pathological finding on ultrasound of kidneys). All the patients with cirrhosis and HRS had mild to large ascites. Creatinine values were >130 μmol, corresponding to type II HRS [21].

The study was conducted in accordance with the ethical standards of the Committee for Experiments on Humans with compliance of the Ethical Committee after obtaining consent from every patient.

General biochemical parameters were determined by biochemical IFCC methods (kinetic spectrophotometric methods on multi-channeled biochemical OLYMPUS-AU 680 analyzer). Determining MDA plasma concentration was obtained by Ledwozyw et al. [23] method. This method is a colorimetric determination of MDA using thiobarbituric acid. Determining GSH plasma concentration was obtained using the Sedlak and Lindsay method [24], a colorimetric determination of GSH based on Elman reagent reaction (2-nitrobenzene acid) and SH group of GSH. Additionally, determining GST plasma activities was done with the Habig method [25] that is based on the GST capability to catalyze conjugation reaction of GSH with substrate 1-chlor 2.4-dinitrobenzene (CDNB). Determination of NO_2_ + NO_3_ concentration was performed using the Navaro-Gonzalvez et al. [26] semiautomated method, based on diazotization of sulfanilic acid.

Data are shown as mean values and their standard deviations (95% CI for mean), where the differences in groups were determined with the application of ANOVA test. Furthermore, *post hoc* analysis, the Dunnett’s T3, and Tukey test were used. Statistically important difference was accepted with risk of *p* < .05. Ratio between investigated variables was determined by linear regressive analysis and goodness of fit analysis as well as using Pearson’s coefficient of correlation. Interconnection of the followed-up biochemical markers with the development of HRS in patients with alcohol-induced liver cirrhosis was investigated with binary logistic regression analysis (Enter model).

## Results

Basic demographic and laboratory analyses are shown in Table 1. A significant liver damage was detected in the group of patients with HRS as indicated by de Ritis coefficient. The damage was significantly higher in patients with HRS compared to the patients without HRS and much higher compared to healthy subjects.

**Table 1.** Basic demographic, clinical, and biochemical characteristics in examined groups and in healthy controls.

	Cirrhosis with HRS type II	Cirrhosis	Healthy controls
Males/females	48/0	32/0	42/0
Age (years)	55.83 ± 12.41*	50.58 ± 11.08*	51.13 ± 23
AST/ALT	3.29 ± 1.72*	1.88 ± 0.63*	1.12 ± 0.21
Albumin (g/L)	24.78 ± 3.59*	29.75 ± 4.33*	40.28 ± 3.78
CRP (mg/L)	15.12 ± 4.39*	9.73 ± 2.51*	2.45 ± 0.64
Urea (mmol/L)	9.92 ± 1.90*	7.23 ± 2.40	3.41 ± 1.35
Creatinine (µmol/L)	167.76 ± 28.99*	89.85 ± 17.80	73.19 ± 15.59

The study included a total of 80 patients with cirrhosis and 42 healthy individuals. The results are presented as mean values with standard deviations using ANOVA test, *post hoc* analysis, Dunnett’s T3, and Tukey test.

* Statistically significant difference was acceptable at the risk level of *p* < .05.

Synthetic liver function was significantly reduced in patients with HRS and in patients without HRS compared to healthy subjects. This was observed through the serum albumin level. Excretory liver function was significantly reduced in patients with HRS compared to the patients with HRS and especially in the healthy subjects. This was observed through the total level of bilirubin and through a significant increase of indirect bilirubin. The parameters of renal failure, urea and creatinine were significantly increased in patients with HRS compared to patients without HRS and especially in healthy subjects.

**Figure 1.**
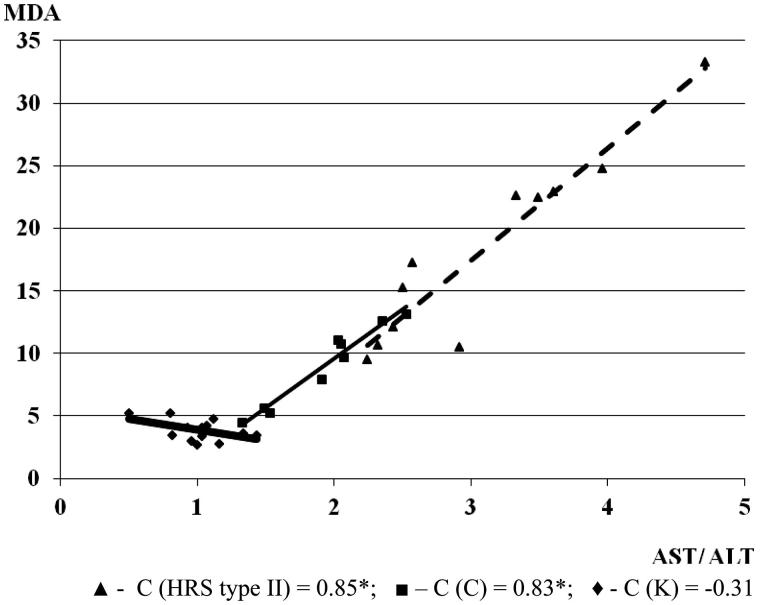
The correlation between MDA and AST/ALT in the experimental groups (the correlation between examined parameters was determined by linear regression analysis and ‘goodness of fit’ analysis, as well as by the Pearson’s coefficient of linear correlation). MDA is expressed in µmol/L.

Figure 1 shows a correlation between MDA and AST/ALT, in groups with cirrhosis with and without HRS. In cirrhosis, the level of MDA increased with the increase of ratio AST/ALT (*C* = 0.83; *p < .05). With the progression of cirrhosis and the development of HRS in the condition of the ongoing renal failure, the correlation of MDA and AST/ALT increased (*C* = 0.85). This could be the indicator of oxidative stress, liver damage and progression of liver cirrhosis with the development of renal failure (Figure 1). Contrary, there was a negative correlation between MDA and CRP level in the control group.

**Figure 2.**
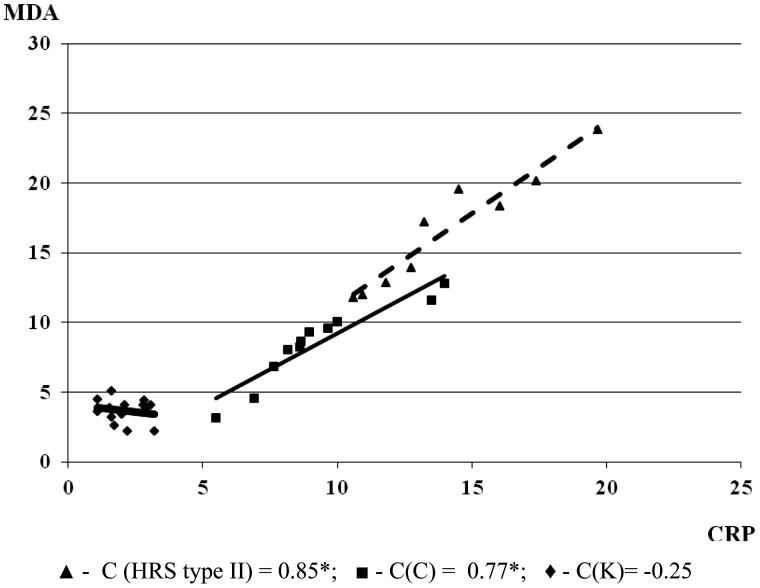
The correlation between MDA and CRP in experimental groups (the correlation between examined parameters was determined by linear regression analysis and ‘goodness of fit’ analysis, as well as using Pearson’s coefficient of linear correlation). MDA is expressed in µmol/L and CRP in mg/L.

In cirrhosis, the MDA level increased with the rise of CRP (*C* = 0.77). With the progression of cirrhosis and the development of HRS in renal failure, the interdependence of MDA and CRP positively correlated (*C* = 0.85; *p < .05). This could also indicate that oxidative stress is important for the development of liver inflammation which significantly influences the damage of liver function and progression of renal failure (Figure 2).

**Figure 3.**
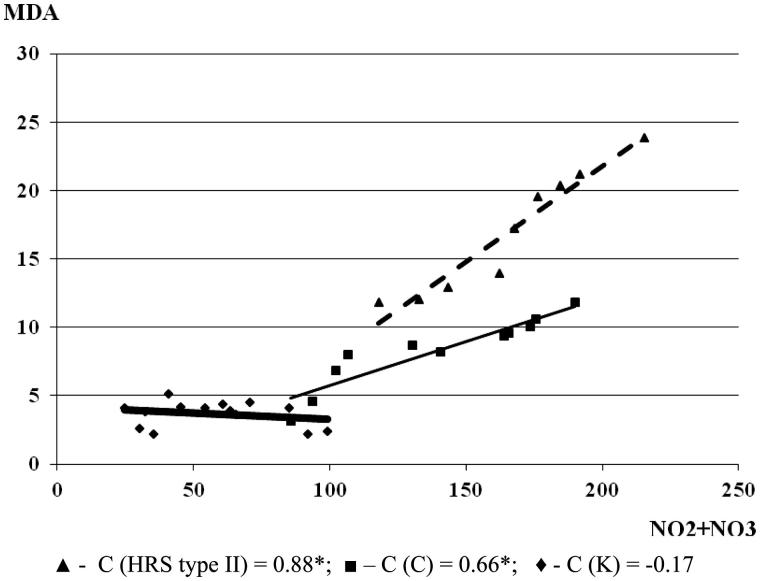
The correlation between MDA and NO_2_ + NO_3_ in the experimental groups (the correlation between examined parameters was determined by a linear regression analysis and ‘goodness of fit’ analysis, as well as by Pearson’s coefficient of linear correlation). MDA and NO_2_ + NO_3_ are expressed in µmol/L.

In cirrhosis, the level of MDA increased with the rise of NO_2_ + NO_3_ (*C* = 0.66) level. With the progression of cirrhosis and HRS development in the condition of renal failure, the interdependence of MDA and NO_2_ + NO_3_ positively correlated (*C* = 0.88; *p < .05). This could indicate that lipid peroxides are interdependent with the iNOS in the pathogenesis of liver diseases, which may significantly influence the inflammatory damage of the liver function and progression of renal failure (Figure 3).

**Figure 4.**
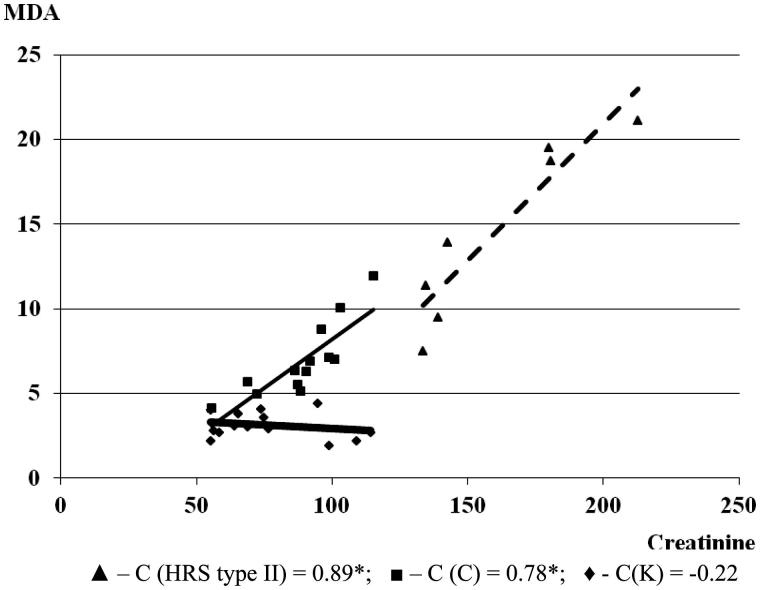
The correlation between MDA and creatinine in the experimental groups (the correlation between examined parameters was determined by a linear regression analysis and ‘goodness of fit’ analysis, as well as by Pearson’s coefficient of linear correlation). MDA and creatinine are expressed in µmol/L.

A significant negative correlation between MDA and creatinine concentration was found in the control group. In cirrhosis, the level of MDA in the serum increased with the growth of creatinine concentration (*C* = 0.78; *p < .05). With the progression of cirrhosis and the development of HRS in the condition of severe renal failure, the interdependence of MDA and creatinine increased (*C* = 0.89). This would indicate that the level of MDA can influence the damage of liver function and further progression of renal failure (Figure 4).

**Figure 5.**
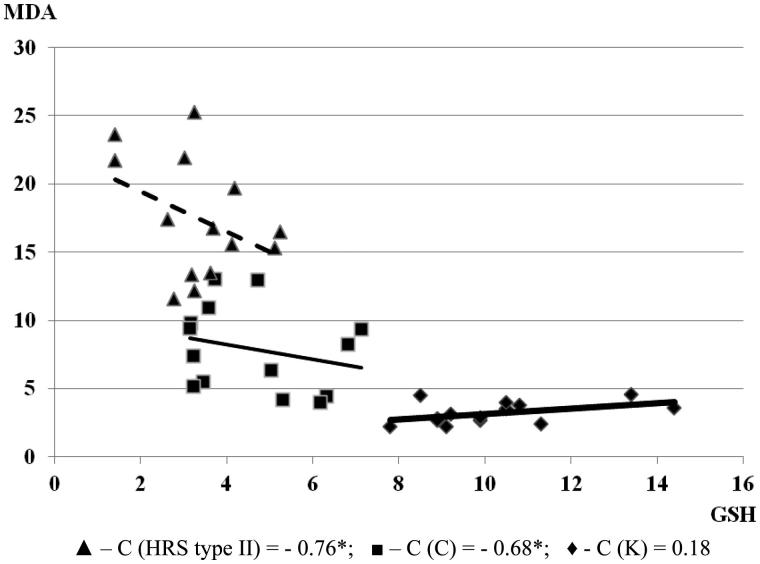
Correlation of MDA and GSH in examined groups (the relationship between investigated parameters was determined by linear regression analysis and ‘goodness of fit’ analysis, as well as using Pearson’s coefficient of linear correlation). The MDA is expressed in µmol/L and glutathione is expressed in µmol/L.

**Figure 6.**
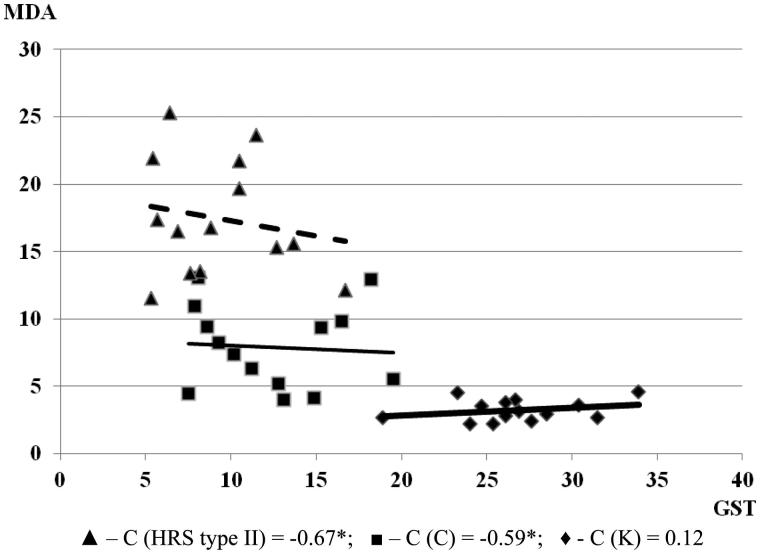
Correlation of MDA and GST in examined groups (the relationship between examined parameters was determined using linear regression analysis and ‘goodness of fit’ analysis, as well as using Pearson’s coefficient of linear correlation). MDA is expressed in µmol/L and glutathione S-transferase is expressed in U/L.

The lowest values of glutathione and GST were registered in the group with HRS type II. A significant negative correlation was found between MDA and both glutathione and GST values in the groups with HRS. In cirrhotic patients, serum MDA level increased along with a decrease of GSH values (*C* = −0.68, *p* < .05), and a decrease of GST values (*C* = −0.59; *p* < .05). With the progression of cirrhosis and HRS development, as well as manifested renal failure, a negative correlation between MDA and GSH (*C* = −0.76; *p* < .05) and negative correlation between MDA and GST (*C* = −0.67*; *p* < .05) increased (Figures 5 and 6). A positive correlation was found between MDA and both GSH and GST values in the control group. Obtained MDA, glutathione and GST values in all three groups are illustrated in Table 2. The MDA level was significantly elevated in the groups with cirrhosis and HRS, higher in comparison to the group with cirrhosis without HRS and the control group. This may suggest that LP and inflammation within hepatocytes, Kupffer cells, and hepatic sinusoidal vascular endothelial and glomerular cells, along with the decline and the exhaustion of AOS, result in progressive hepatic and renal impairment.

**Table 2.** Parameters of oxidative stress and antioxidative defense in examined groups and in healthy controls.

	Cirrhosis with HRS type II	Cirrhosis	Healthy controls
MDA (µmol/L)	17.28 ± 4.34*	7.94 ± 3.05*	3.36 ± 0.76
GSH (µmol/L)	3.29 ± 1.24*	4.51 ± 1.60*	10.86 ± 1.85
GST (U/L)	9.19 ± 3.43*	12.21 ± 4.27*	26.41 ± 3.78

Determination of MDA plasma concentration was performed using the method of Ledwozyw et al. Determination of GSH plasma concentration was done using the method of Sedlak and Lindsay, and determination of GST plasma activity was performed by the method of Habig. The results are presented as mean values with standard deviations using ANOVA test, *post hoc* analysis, Dunnett’s T3, and Tukey’s test.

* Statistically significant difference was acceptable at the risk level of *p* < .05.

**Figure 7.**
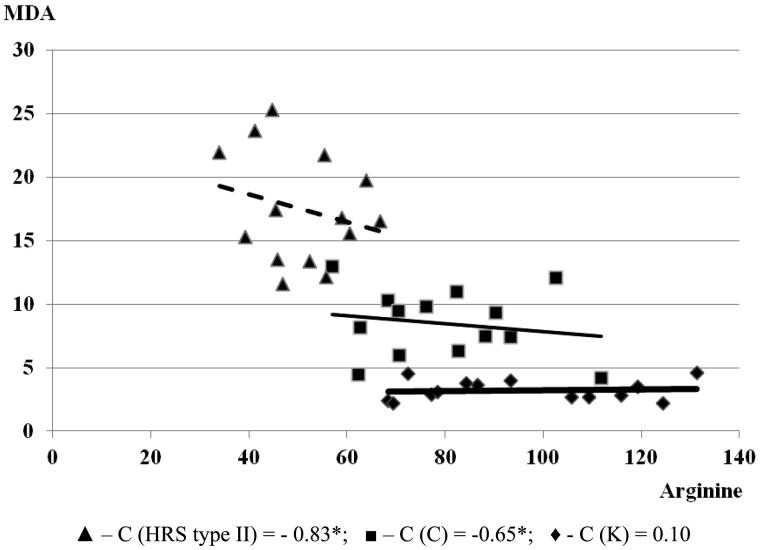
Correlation of MDA and arginine in examined groups (the relationship between examined parameters was determined using linear regression analysis and ‘goodness of fit’ analysis, as well as using Pearson’s coefficient of linear correlation). MDA is expressed in µmol/L. L-arginine was expressed in µmol/L.

Table 3 illustrates L-arginine and NO_2_ + NO_3_ values in investigated study groups. The lowest L-arginine values were registered in the group with HRS type II. A significant negative correlation was found between the MDA levels and L-arginine values in the group with HRS (*C* = −0.83, *p* < .05) (Figure 7). The level of NO_2_ + NO_3_ was significantly elevated (two- and three-fold) in the groups with cirrhosis with and without HRS in comparison to the controls. A significant negative correlation was found between the MDA and NO_2_ + NO_3_ levels in the group with cirrhosis without HRS (*C* = −0.66*, *p* < .05). The correlation between the MDA and NO_2_ + NO_3_ levels increased (*C* = −0.88, *p* < .05) with the progression of cirrhosis and HRS development. It could suggest the L-arginine metabolites participation in pathogenesis of renal and hepatic impairment in chronic alcoholism, besides oxidative stress, and inflammation.

**Table 3.** L-arginine and NO_2_ + NO_3_ in in examined groups and in healthy controls.

	Cirrhosis with HRS type II	Cirrhosis	Healthy controls
L-arginine (µmol/L)	52.36 ± 11.14*	79.28 ± 16.15*	97.26 ± 21.72
NO_2_ + NO_3_ (µmol/L)	173.79 ± 30.59*	142.95 ± 31.16*	61.19 ± 18.73

The determination of L-arginine level was performed by the use of HPLC method. NO_2_ + NO_3_ concentration was determined using the method of Navaro-Gonzalvez. The results are presented as mean values with standard deviations using ANOVA test, *post hoc* analysis, Dunnett’s T3, and Tukey’s test.

* Statistically significant difference was acceptable at the risk level of *p* < .05.

## Discussion

Lipid peroxides and L-arginine metabolites can influence liver and kidney damage in chronic alcoholism. Our previous research also pointed out such findings about ADMA and SDMA. Obtained results showed that the level of MDA was significantly increased in patient groups with cirrhosis with or without HRS in comparison to the control group. Results also showed that the level of MDA positively correlates with the level of liver damage, that is, with De Ritis coefficient in groups with cirrhotic patients. Disparate values in the level of MDA in groups with or without HRS, correlate with the level of fibrosis and the level of inflammation in liver cirrhosis [27]. Unlike our results, a study by Vuppalanchi [28] showed that MDA correlated poorly with hepatic tissue oxidative stress. Generally, alcohol is metabolized in the liver by ADH pathway. Chronic consumption of alcohol enhances production of acetaldehyde reduces NAD in the cytosol and mitochondria, as well as the activity of MEOS and cytochrome P 4502E1 enzyme. Activity of cytochrome P 4502E1 enzyme permanently produces significant amounts of free radicals, acetaldehydes, and MDAs. MDA as highly reactive binds to lipoproteins of hepatocyte membrane, promoting membrane damage [7]. Accumulation of reduced NADP leads to hepatocyte hypoxia and ATP reduction, required for the synthesis of glutathione, a major intracellular antioxidant. Aldehydes and lipid peroxides stimulate hepatic fibroproliferation. In cirrhosis, during the hepatic fibrogenic process, MDA may act in three ways: at gene expression levels (regulates gene expression for pro-collagen synthesis), by NF-κB macrophages (transforms Ito cells into myofibroblastic cells for the production of collagen and other components of extracellular matrix), and functions as an antigen in autoimmune response, together with acetaldehyde-protein adducts [29]. In the hepatic fibrogenic process, expression of RAGE receptors is increased in hepatic stellate cells. MDA interaction with RAGE receptors stimulates the transcription of genes for cytokines and growth factor. Proinflammatory cytokines increase the expression of adhesive molecules, as well as permeability of blood vessels, thus producing toxic effects [30]. Malondialdehyde-acetaldehyde (MAA) adducts may cause hepatocyte apoptosis and necrosis, initially perivenously in the centrilobular areas of the liver [31]. Similar results were obtained in a study by Aleynik et al., showing that the MDA levels increase in proportion with alcoholic cirrhosis progression [32]. Considering the inflammatory process, the result show persistently elevated CRP levels in patients with cirrhosis. The progression of cirrhosis and the development of HRS type II may result in positive correlation between MDA and CRP. Mortensen et al. highlighted the importance of inflammation in the pathogenesis of alcoholic cirrhosis [33]. MDA, along with AOPP and AGE, through the interaction with RAGE receptors, regulates gene expression for the synthesis of CRP. Similar results were reported in a study by Kirkhama et al., showing that the involvement of both oxidative stress and inflammation progress into end-stage liver failure [34]. In conditions of cumulative oxidative stress and inflammation, cirrhotic liver redirects a normal metabolic arginine path in the direction of synthesis of pathological metabolites, peroxynitrates. The group with cirrhosis exhibited lower values of L-arginine and negative correlation between L-MDA and L-arginine. Chronic alcoholic liver disease, oxidative modifications of liver parenchymal components may reduce L-arginine production and intrahepatic vasodilatory NO synthesis [35]. Liver fibrosis may increase intrahepatic vascular resistance and may stimulate the development of portal hypertension and its complications [36]. In cirrhotic liver a reduction in NO production and peroxynitrites increase on the one hand, and increased vasoconstrictive activity of endothelin and angiotensin on sinusoidal endothelial cells on the other hand, aggravate portal hypertension [37]. Results also showed a significant correlation of MDA and NO_2_ + NO_3_. In conditions of endotoxemia, the liver macrophage and monocytes through CRP cytokines stimulate iNOS to increasingly produce NO and peroxynitrites. Peroxynitrite ion reacts with fatty acids of lipoprotein membranes leading to theiroxidative modification which potentiates hepatocytes damage and other complications of cirrhosis [38]. A significant negative correlation between the MDA and GSH, as well as between the MDA and GST was found in patients with cirrhosis. The role of MDA and AOS enzymes in the pathogenesis of alcoholic liver disease has also been proved by Arteelet et al. [39]. It could indicate that MDA level may impair liver function and may stimulate the development of complications. Continuous and cumulative oxidative stress in alcoholic patients prevails over synthesis and regeneration of sufficient amounts of reduced glutathione, reduces GSH and glutathione-dependent enzymes activities, thus stimulating liver impairment [40].

In advanced decompensated cirrhosis, systemic hypotension, local reduction of vasodilatory substances, and potential presence of bacterial endotoxins may result in a profound renal hypoperfusion. In patients with HRS, liver disease progression and transition to the decompensated stage of the disease due to the development of mild-to refractory ascites aggravate renal retention of sodium [41,42]. In patients with HRS contributing factors for renal failure, GFR reduction may also include the loss of renal blood flow autoregulation, the contraction of mesangial cells stimulated by endothelin, the activation of inflammatory markers leukotriene and thromboxane A, followed by a reduction of NO synthesis in liver and reduced sensitivity of the receptors on glomerular endothelial cells to NO [4,43].

In patients with HRS type II, a dysfunction of immunoinflammatory system develops due to the retention of urine and constant recovery of blood contact with endotoxins in a catheter. Endotoxines from catheter-related bacteria stimulate macrophages to produce more interleukin 6 and TNF-α, parameters of the inflammatory response in serum, thus resulting in additional systemic blood volume reduction. In patients with HRS, endotoxemy and inflammation with increased production of both leukotrienes C4 and D4 and F2-isoprostane may lead to a decrease in GFR [44,45]. The evaluation of renal function levels of urea and creatinine was determined. In the HRS type II group, creatinine level was as twice as high when compared to the level in cirrhosis and higher in comparison to the control group level. Results also showed that MDA level significantly correlates with the creatinine level in the HRS patient group. Correspondingly, the results of the Locatelli et al.’s [46] study indicate a correlation of MDA with creatinine. Urine retention and endotoxemia intensify liver damage and the development of chronic renal failure. The ramp-up of lipid peroxides in uremic patients is explained by a constant interaction of blood with biocompatible catheter membrane that induces circulating neutrophils to produce ROS (‘respiratory burst ROS’) [47]. Mediated by ROS, proinflammatory cytokines such as IL-6, IL-8, IL-1b, chemokines, TNF-α, eicosanoids and reactive NO compounds synthesize in neutrophils. Proinflammatory cytokines alarm the liver to produce CRP, cytokines, fibrinogen, and other inflammatory factors which induce liver inflammation and kidney parenchyma [48]. In neutrophils, the plasma membrane NADPH-oxidase stimulates the increased production of peroxynitrite anion (nitrosative stress) [49]. Myeloperoxidase catalyzes reaction of hypochlorite acid synthesis. Hypochlorite acid and peroxynitrite anion react with fatty lipid acids, thus significantly increasing the level of lipid peroxides. Hypochlorite acid is a strong antioxidant that dissolves the parenchyma matrix and potentiates renal damage [50]. Our results are consistent with the results of Branden et al. [51], but differ from the results of the Zuwała-Jagiełło et al. [16] study who have reported that with the progression of renal failure, MDA shows a steady stream.

In uremic patients, chronic inflammatory condition may induce damage of vascular endothelium. Apart from pro-oxidative and pro-inflammatory process, MDA may also contribute to pro-atherogenic condition of vascular endothelium in uremic patients. MDA reacts with phospholipids of the membranes and oxidatively modifies LDL cholesterol [52]. Peroxynitrites perform nitration of LDL proteins and shore-up an accelerated atherosclerosis, portal hypertension, and complications [53]. Modulated LDL particles accumulate cholesterol in macrophages ensuing high incidence of atherosclerotic plaque development. In patients with HRS, accumulation of lipid peroxides is a key event in pathogenesis of accelerated atherosclerosis that remains the leading cause of morbidity and mortality [54,55]. In the HRS type II groups, GSH and GST activities are significantly lower in comparison to their activities in cirrhosis and lower in comparison to the control group. A significant negative correlation between the MDA and GSH as well as between the MDA and GST was found in the groups with HRS type II. The accumulation of lipid peroxides results in glutathione exhaustion and detoxification activity of GST enzymes. Glutathione can directly neutralize MDA, peroxynitrites and other oxidative cellular molecules, and it can indirectly, as a coenzyme of GST and GPX, detoxify toxic compounds. Significant glutathione reduction in the group with HRS is also explained by the reduction of GPX-isoenzyme 3 (with GSH as a coenzyme of GPX) that is produced only in kidneys [56]. GST transforms MDA into a water-soluble product excreted by the kidneys. Continuous oxidative stress, namely the MDA increase and GST decrease, additionally reduces renal function in alcoholic patients with HRS. The accumulation of lipid peroxides induces electron leakage in the respiratory chain. Disorder of the membrane potential generates a mitochondrial dysfunction and interruption of ATP-dependent glutathione synthesis. A disruption of ATP synthesis intensifies cellular apoptosis both in the liver and kidney and tissue degradation [57,58]. Mitochondrial dysfunction advances O_2_^−^ and NO^−^ anion production [59]. A reduction in GFR is a major abnormality in renal function in cirrhosis. In patients with cirrhosis, plasma lipid oxidation is induced by peroxynitrites which initiate glomerular endothelial injury. In patients with HRS type II, the value of L-arginine was the lowest, and there was a significant correlation between the MDA and L-arginine. The pronounced oxidative stress and inflammation followed by the production and accumulation of pathological L-arginine metabolite SDMA increased within renal endothelium in renal failure. SDMA competes with L-arginine for NO synthesis via Y^+^ transporters at the level of glomerular endothelial membrane [60]. High levels of SDMA and peroxynitrites may inhibit L-arginine absorption, what may induce L-arginine deficiency in renal endothelial cells, thus stimulating renal perfusion impairment [61]. In comparison with other segments of the nephron, tubular epithelial cells do not have the capacity to synthetize GSH, so they suffer most from mitochondrial disfunction [17]. Prolonged renal hypoperfusion, medullary hypoxia, as well as frequent infections, may contribute to the development of glomerulosclerosis and interstitial fibrosis. This results in the terminal stage of renal failure [62]. Urine retention, endotoxemy as well as lipid peroxides and peroxynitrites with exhausted AOS activities result in progressive tissue degradation, systemic endothelial dysfunction, end-stage renal disease and the development of the vascular collapse [63].
